# Inflammatory bowel disease increases the levels of albuminuria and the risk of urolithiasis: a two-sample Mendelian randomization study

**DOI:** 10.1186/s40001-023-01128-0

**Published:** 2023-05-12

**Authors:** Hao Wu, Peng Liu, Siming Gong, Xiaoming Liu, Michael A. Hill, Zhenguo Liu, Meihua Xu, Canxia Xu

**Affiliations:** 1 Department of Gastroenterology, Third Xiangya Hospital, Central South University, Tongzipo Road 138, Changsha, 410013 Hunan China; 2 Department of Orthopaedics, Xiangya Hospital, Central South University, Changsha, China; 3 Dalton Cardiovascular Research Center, University of Missouri, Columbia, MO USA; 4 Center for Precision Medicine, University of Missouri School of Medicine, Columbia, MO USA; 5 Department of Gastroenterology, Xiangya Hospital, Central South University, No.87 Xiangya Road, Changsha, 410008 Hunan China

**Keywords:** Crohn’s disease, Ulcerative colitis, Urine albumin–creatinine ratio, Urolithiasis, Mendelian randomization

## Abstract

**Background:**

Alterations in kidney function and increased risk of kidney diseases in patients with inflammatory bowel disease (IBD) have been reported, but the causal relationship remains unclear. Herein, Mendelian randomization was employed to identify the causal effect of inflammatory bowel disease on kidney function and the risk of chronic kidney disease (CKD), urolithiasis, and IgA nephropathy.

**Methods:**

The International Inflammatory Bowel Disease Genetics Consortium provided the summary-level genome-wide association study (GWAS) data that correlates with Crohn's disease (CD) and ulcerative colitis (UC). GWAS data for estimated glomerular filtration rate from serum creatinine (eGFRcrea), urine albumin–creatinine ratio (uACR), and CKD were obtained from the CKDGen Consortium, and GWAS data for urolithiasis were obtained from the FinnGen consortium. The summary-level GWAS data for IgA nephropathy were obtained from the meta-analysis of UK-biobank, FinnGen, and Biobank Japan. Inverse-variance weighted was used as the primary estimate. Furthermore, the Steiger test was used to validate the direction of causality.

**Results:**

The inverse-variance weighted data revealed that genetically predicted UC significantly increased uACR levels, while genetically predicted CD significantly increased the risk of urolithiasis.

**Conclusions:**

UC increases the levels of uACR, and CD increases the risk of urolithiasis.

**Supplementary Information:**

The online version contains supplementary material available at 10.1186/s40001-023-01128-0.

## Introduction

Inflammatory bowel disease (IBD), including Crohn’s disease (CD) and ulcerative colitis (UC), has become globally significant in the twenty-first century [1]. IBD is a systemic disease that is associated with significant extraintestinal manifestations including hepatobiliary disease, cardiovascular disease, arthropathy and arthritis, and skin disease, in addition to the gut and gastrointestinal tract [2]. Recent studies have demonstrated that patients with IBD are also at risk for kidney-related extraintestinal manifestations other than IBD drug-induced nephrotoxicity [3, 4]. However, a causal relationship between IBD and kidney function or disease remains unclear.

A retrospective cohort study of over 80,000 individuals showed that IBD is associated with an increased risk of chronic kidney disease (CKD) [5]. A Swedish population-based cohort study demonstrated a notable association between IBD and IgA nephropathy both prior to and following the diagnosis of IgA nephropathy. Moreover, patients with IgA nephropathy with IBD were found to have a higher risk for end-stage renal disease (ESRD) than those without IBD [6]. Indeed, IBD susceptibility loci were shared with IgA nephropathy (*HLA-DQ/DR, CARD9, HORMAD2*), which may explain why these two conditions tend to have a higher incidence of co-occurrence than expected by chance [7]. Measurement of urine albumin–creatinine ratio (uACR) is a reliable method to predict renal events in patients with nephropathy. Indeed, several small-sample studies have reported that proteinuria is an apparent renal extraintestinal manifestation of IBD and could potentially function as a marker of disease activity in IBD [8, 9]. In addition, a nationwide Danish cohort study demonstrated that individuals diagnosed with IBD faced twice the risk of developing urolithiasis [10]. However, these observational studies have limitations, such as confounding bias.

Mendelian randomization (MR) is an approach that generates more reliable evidence for causal relationships using genetic variants that are robustly linked with exposures, which could avoid confounding bias inherent in observational studies. Previous MR studies have reported on numerous risk factors associated with IBD, as well as the causal relationship between IBD and other diseases [11–13]. Nevertheless, a causal relationship between IBD and kidney function or disease has not been demonstrated yet. Two-sample MR analysis is an advanced version of the MR technique that permits the use of summary data from genome-wide association studies (GWAS) without the need for direct analysis of individual-level data. In the present study, a two-sample MR study was conducted to identify the causality between IBD (including UC and CD) and kidney function/disease (including eGFRcrea, uACR, CKD, urolithiasis, and IgA nephropathy) using available large-scale GWAS data.

## Methods

The present study was conducted in accordance with the “STROBE-MR” statement [14].

### Data sources for Crohn’s disease, and ulcerative colitis

The International Inflammatory Bowel Disease Genetics Consortium (IIBDGC, https://www.ibdgenetics.org/) provided the summary-level GWAS data correlated with UC and CD. The GWAS data for UC included 13,768 patients and 33,977 controls, and the GWAS data for CD included 17,897 patients and 33,977 controls.

### Data sources for estimated glomerular filtration rate from serum creatinine, urine albumin–creatinine ratio, urolithiasis, IgA nephropathy, and chronic kidney disease

The CKDGen Consortium (https://ckdgen.imbi.uni-freiburg.de/) provided the summary-level GWAS data associated with eGFRcrea, uACR, and CKD. Meta-analysis of the GWAS data for eGFRcrea included 567,460 persons of European ancestry. For individuals 18 years or younger, the Schwartz formula was used to calculate eGFRcrea, while adults had their eGFRcrea calculated according to the Chronic Kidney Disease–Epidemiology Collaboration (CKD–EPI) equation. Meta-analysis of GWAS data for uACR included 547,361 subjects of European ancestry. uACR was evaluated in mg/g, calculated as urinary albumin (mg/l) divided by urinary creatinine (mg/dl), and then multiplied by 100. Meta-analysis of GWAS data for CKD included 64,164 European ancestry cases and 561,055 controls of European ancestry. CKD was defined as an eGFRcrea below 60 ml/min/1.73 m^2^. The details of the CKDGen Consortium have been introduced by Teumer et al. and Wuttke et al. [15, 16]. The summary-level GWAS data related to urolithiasis were acquired from the FinnGen consortium, Freeze 7 (8060 cases and 301,094 controls, https://r7.finngen.fi/pheno/N14_UROLITHIASIS). The definition of urolithiasis was based on ICD-10 (N20–N23). The summary-level GWAS data for IgA nephropathy were acquired from the meta-analysis of FinnGen, UK-biobank, and Biobank Japan (https://www.ebi.ac.uk/gwas/studies/GCST90018866). Meta-analysis of GWAS data for IgA nephropathy included 15,587 cases of European ancestry, 462,197 European ancestry controls, 71 cases of East Asian ancestry, and 175,288 East Asian ancestry controls.

### Selection of genetic instruments

The genetic instruments were chosen based on the following criteria: (1) single nucleotide polymorphisms (SNPs) associated with UC or CD with genome-wide significance (*p* < 5 × 10^–8^) and (2) SNPs for the UC or CD were not in linkage disequilibrium (LD) via clumping process (*r*^2^ < 0.001 within a window size of 10,000 kb). We identified 86 SNPs related to UC and 115 SNPs related to CD from European-ancestry participants of the IIBDGC. To test for weak instrumental variable bias, we calculated *F* statistics using the formula *F* = *R*^*2*^(*n-k-1*)*/k(1-R*^*2*^), where *R*^*2*^ is the proportion of the variance of the exposure explained by the instrumental variables, *n* sample size, and *k* number of genetic variants). If the* F* statistics for the instrument–exposure association is much greater than 10, the possibility of weak instrumental variable bias is low. To remove the kidney-related SNPs (eGFRcrea, uACR, urolithiasis, IgA nephropathy, and CKD), we used a threshold of 5 × 10^–8^, and then applied MR–PRESSO to eliminate any outliers before conducting each MR analysis.

### Mendelian randomization analyses

The random-effect inverse variance weighted (IVW) method was performed as the major approach, while MR–Egger and weighted median (WM) methods were used to improve the IVW estimates by providing more robust estimates over a broader range of scenarios (wider confidence intervals with lower efficiency). The IVW method is an efficient analysis when all genetic variants are valid instrumental variables [17]. The MR–Egger method can assess whether genetic variants have pleiotropic effects on the outcome that are different from zero on average, and it can also provide a reliable estimate of the causal effect, even under the weaker InSIDE (Instrument Strength Independent of Direct Effect) assumption [18]. The WM method permits the utilization of invalid instrumental variables while requiring that at least 50% of the instrumental variables used are valid [19]. The Cochran’s Q test was utilized to assess heterogeneity. The Egger intercept test and leave-one-out (LOO) analyses were utilized to evaluate horizontal pleiotropy. For significant estimates, PhenoScanner V2, a database of human genotype–phenotype associations, was used to determine whether the SNPs were linked to potential risk factors, such as obesity, smoking, hypertension, diabetes, and disorders of mineral metabolism. In addition, the Steiger test was used to validate the direction of observed causalities [20].

### Statistics

All analyses were conducted by the packages TwoSampleMR (version 0.5.6) and MRPRESSO (version 1.0) in R platform (version 4.2.2). *P* < 0.05 were considered statistically significant.

## Results

**Fig. 1:**
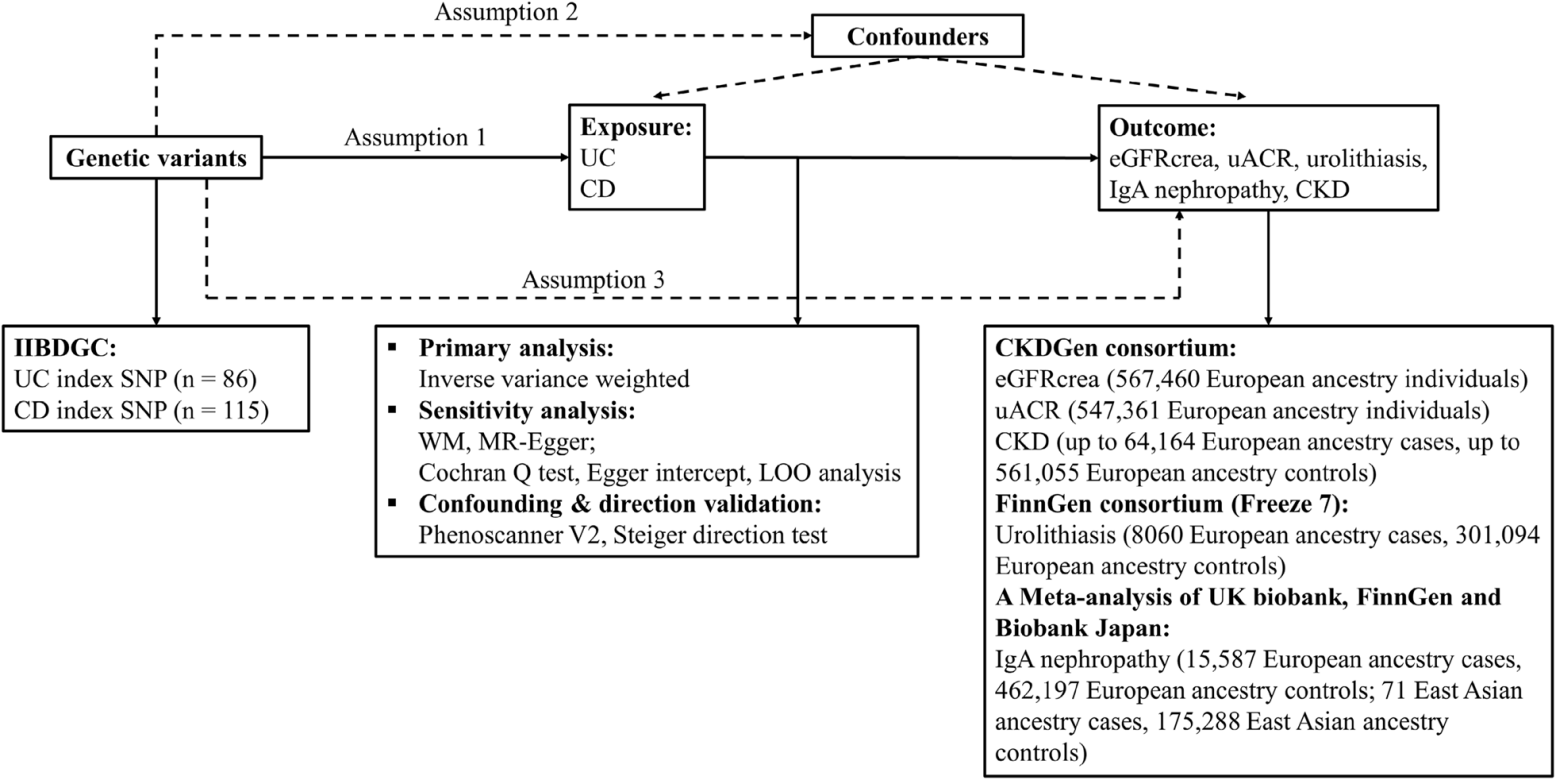
Flowchart of the present Mendelian randomization study. *IIBDGC* International Inflammatory Bowel Disease Genetics Consortium, *SNP* single nucleotide polymorphism, *UC* ulcerative colitis, *CD* Crohn’s disease, *eGFRcrea* estimated glomerular filtration rate from serum creatinine, *uACR* urine albumin to creatinine ratio, *CKD* chronic kidney disease, *WM* weighted median, *LOO* leave-one-out

**Fig. 2:**
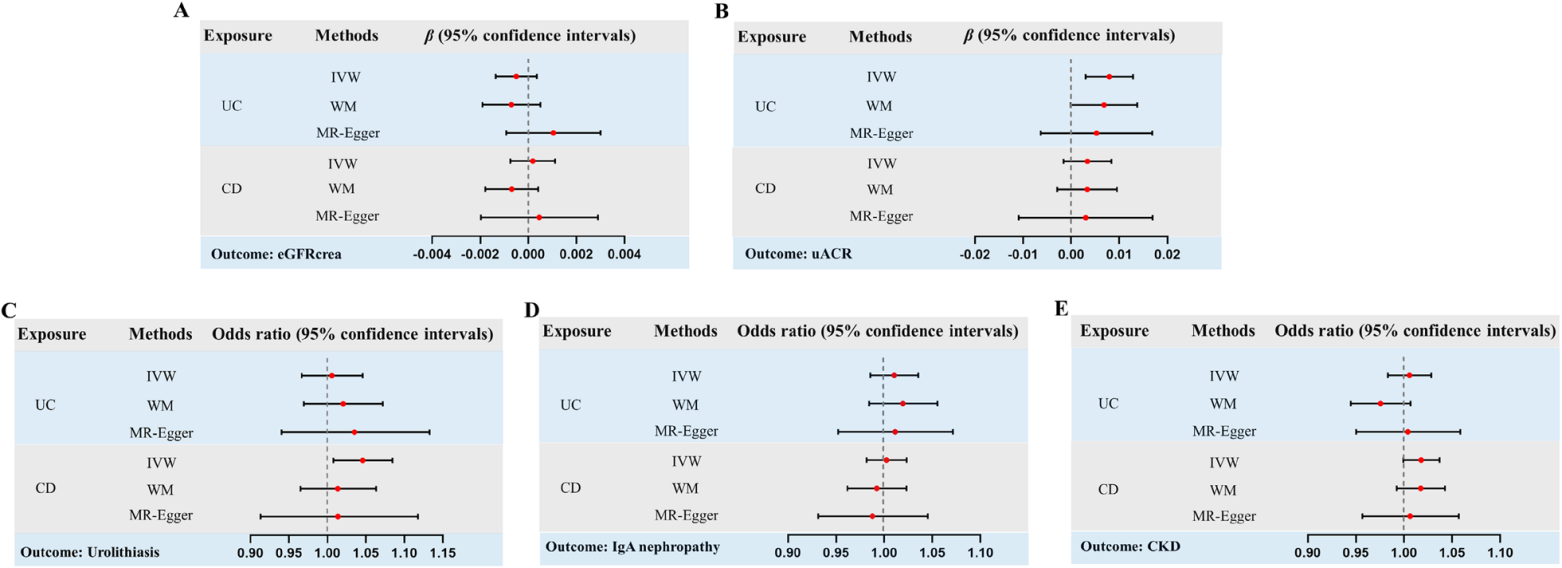
MR estimates from specific methods for evaluation the causal effect of IBD on kidney function. **A** UC and CD on eGFRcrea; **B** UC and CD on uACR; **C** UC and CD on urolithiasis; **D** UC and CD on IgA nephropathy; **E** UC and CD on CKD. *IVW* inverse variance weighted, *WM* weighted median

**Fig. 3:**
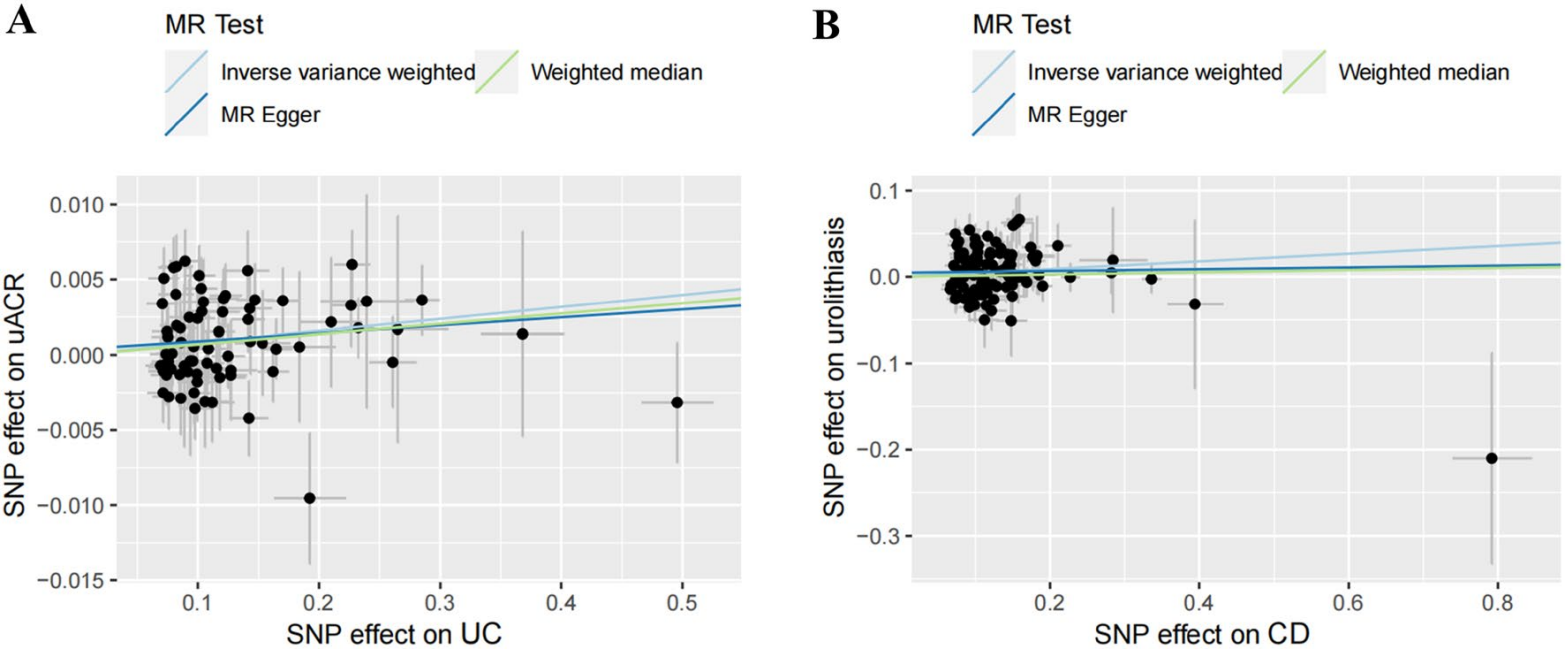
Significant Mendelian randomization association between IBD and kidney function. **A** Scatter plot for genetically predicted UC on uACR; **B** Scatter plot for genetically predicted CD on urolithiasis

A flowchart illustrating the present Mendelian randomization study is shown in Fig. 1. Eighty-six index SNPs (*F* = 77.48) were chosen to genetically predict UC, and 115 index SNPs (*F* = 89.83) were chosen to genetically predict CD. After removing all the outliers by MR–PRESSO, the MR estimates from specific methods of evaluation the causal effect of UC or CD on kidney function are presented in Fig. 2 and Additional file 1: Table S1. The IVW indicated that genetically predicted UC was found to increase the levels of uACR (*β* = 0.008, 95%CI 0.003 to 0.013, *p* = 0.002) (Fig. 2B), but had no causal correlation with eGFRcrea, IgA nephropathy, urolithiasis, and CKD. In addition, the IVW also indicated that genetically predicted CD was found to increase the risk of urolithiasis (OR = 1.046, 95%CI 1.008 to 1.084, *p* = 0.017) (Fig. 2C), but had no causal relationship with eGFRcrea, uACR, IgA nephropathy, and CKD. Scatter plots of significant Mendelian randomization associations are shown in Fig. 3. Additional scatter plots showing non-significant associations are provided in Additional file 1: Figures S1 and S2.

**Fig. 4:**
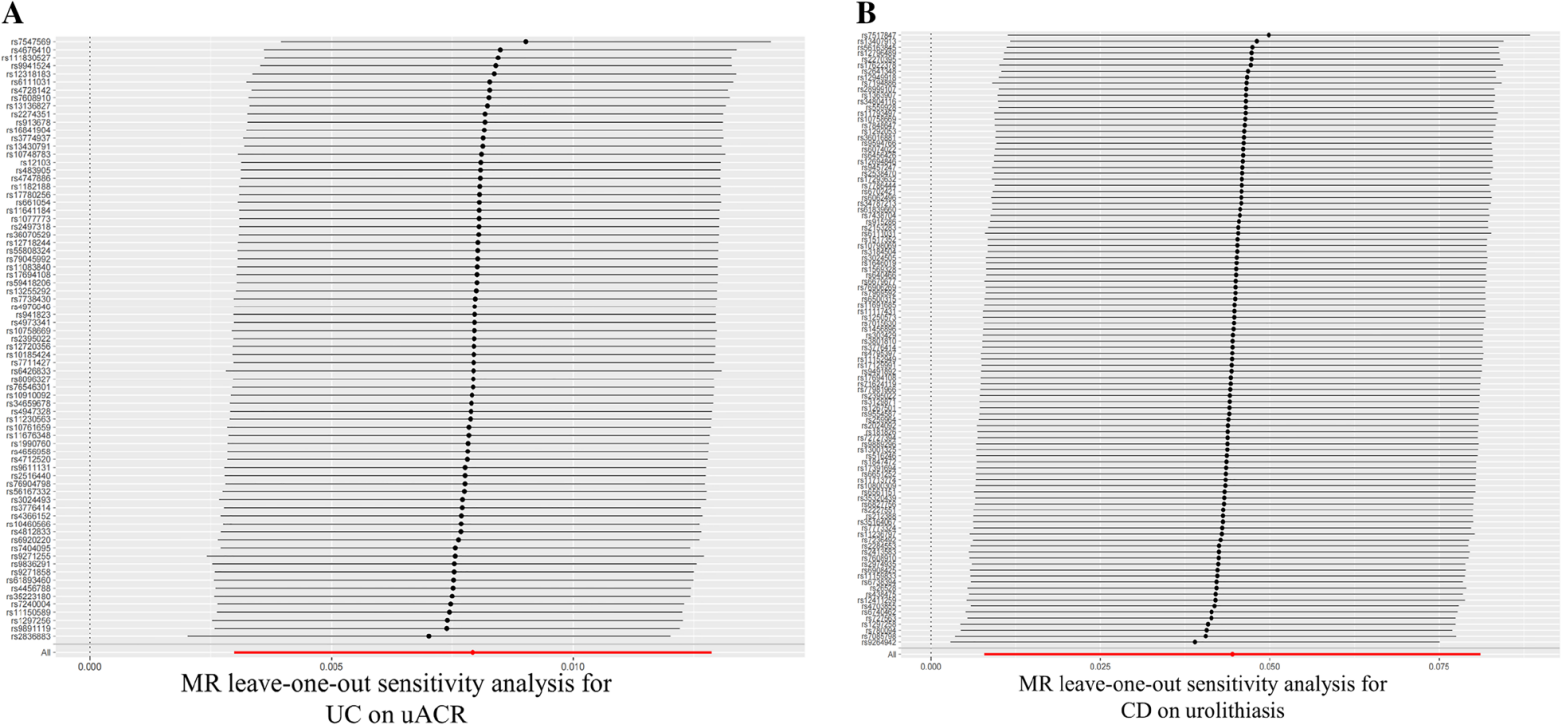
Leave-one-out sensitivity analysis

To assess the robustness of the above outcomes, we conducted sensitivity analyses, which involved leave-one-out analysis, Cochran's Q test, and MR–Egger intercept test. MR–Egger intercept tests demonstrating that there was no horizontal pleiotropy in present study (Table 1). Nonetheless, for significant estimates, heterogeneity was observed in the Q test analyses between UC and uACR (*Q* = 101.31, *P* = 0.019), and CD and urolithiasis (*Q* = 161.52, *P* = 0.0001). Although heterogeneity was found in some results, it did not invalidate the Mendelian randomization estimates, because the present study used random-effect IVW, which may have balanced the pooled heterogeneity. For both significant and non-significant estimates, the leave-one-out analyses are shown in Fig. 4, Additional file 1: Figures S3, and S4. As shown in Fig. 4, the absence of bias from a single SNP suggests that the estimates were not violated. In addition, the Steiger direction test revealed that UC and CD were causes for increased levels of uACR and risk of urolithiasis, separately, but not vice versa (Table 2).

**Table 1:** Sensitivity analysis of the causal association between IBD and kidney function

Exposure	Outcomes	Cochran *Q* test		MR–Egger		MR–PRESSO
		*Q* value	*P*	Intercept	*P*	*P* value
UC	eGFRcrea	98.03	0.022	−0.0002	0.09	0.26
	uACR	101.31	0.019	0.0004	0.62	0.002
	Urolithiasis	105.59	0.011	−0.004	0.54	0.79
	IgA nephropathy	84.62	0.26	−4.49 × 10^–6^	0.99	0.43
	CKD	112.62	0.0078	0.0003	0.92	0.64
CD	eGFRcrea	198.16	9.31 × 10^–10^	−3.86 × 10^–5^	0.81	0.70
	uACR	194.11	2.63 × 10^–8^	4.7 × 10^–5^	0.96	0.19
	Urolithiasis	161.52	0.0001	0.0046	0.48	0.019
	IgA nephropathy	116.99	0.164	0.0022	0.57	0.83
	CKD	146.29	0.0048	0.0017	0.61	0.06

**Table 2:** Steiger direction test from UC to uACR, and from CD to urolithiasis

Exposure	UC	CD
Outcome	uACR	Urolithiasis
Direction	TRUE	TRUE
Steiger *P*	< 0.001	< 0.001

Moreover, the Phenoscanner tool was used to whether test any of the chosen SNPs were correlated with potential risk factors. SNPs rs13407913, and rs17391694 were associated with body mass index and obesity, rs13430791 was associated with diabetes, rs1297256 was associated with hypertensive renal disease, and rs3129871 was related to disorders of mineral metabolism. After removing these SNPs, estimates for UC on uACR and CD on urolithiasis (*β* = 0.008, *p* = 0.003; OR = 1.048, *p* = 0.013) remained consistent with the previous result.

## Discussion

Using large-scale two-sample MR analysis, we systematically evaluated the causal relationship between genetically predicted IBD and kidney function or disease in the present study. Our results show that UC increases the levels of uACR, and CD increases the risk of urolithiasis. These significant results solidify the findings of prior observational studies suggesting that renal and urinary involvement are extraintestinal manifestations of IBD, hence highlighting the existence of the gut-kidney axis.

Proteinuria is central to the diagnosis and management of kidney diseases. Measurement of uACR is a reliable method to predict renal events in patients with nephropathy. The most common type of glomerulonephritis and the primary cause of ESRD in patients with primary glomerular disease is IgA nephropathy [21]. A retrospective study of kidney biopsy in patients with IBD found that the most frequent diagnosis was IgA nephropathy, followed by interstitial nephritis, arterionephrosclerosis, and acute tubular injury. Notably, proteinuria is the common indication for kidney biopsy in patients with IBD [22]. Here, we demonstrate that UC causally increases the levels of uACR, which is consistent with earlier findings in observational studies [8, 9]. However, there was no causal relationship between the IBD and IgA nephropathy in our study. Considering IBD susceptibility loci were shared with IgA nephropathy and the assumption 3 in our MR analysis ruled out the risk factor of an IgA nephropathy outcome, IgA nephropathy probably is a co-occurring disease rather than a secondary manifestation of IBD. Moreover, several studies have demonstrated that there is a positive correlation between the disease activity of IBD and tubulointerstitial damage defined by elevated tubule marker proteins, such as N-acetyl-beta-D-glucosaminidase, alpha-1-microglobulin, and beta-2-microglobulin [8, 23, 24].

The prevalence of urolithiasis in patients with IBD has been shown to be approximately 3–5% [25]. In a recent nationwide cohort study consisting of 75,236 patients with IBD and 767,403 non-IBD individuals, it was discovered that patients diagnosed with IBD had twice the risk of urolithiasis [10]. Similarly, our study found that CD increases the risk of urolithiasis. Indeed, the risk of urolithiasis is higher in CD compared with subjects with UC [26], probably due to frequent ileocolic involvement in CD. Disruption of the gut-kidney axis could play an important role in the development of urolithiasis in patients with IBD. Patients with IBD primarily develop renal stones consisting of uric acid or calcium oxalate. *Oxalobacter formigenes*, a member of the human colonic microbiota, may have an antilithogenic effect in calcium oxalate urolithiasis, through modulation of colonic oxalate transport and secretion [27]. Indeed, patients with both IBD and urolithiasis rarely exhibit *Oxalobacter formigenes* in their stools when compared to controls, which may lead to hyperoxaluria in patients with IBD [28]. In particular, CD patients with urolithiasis had significantly higher levels of urinary oxalate when compared to those without [29]. A recent study based on multi-omics data showed that enteric oxalate levels are elevated in patients with IBD, with highest levels in CD patients with ileocolic involvement. They also demonstrated that microbiota oxalate degradation is decreased in patients with IBD, potentially contributing to the disruption of oxalate homeostasis [30]. Notably, bile acid malabsorption in CD patients with ileocolic involvement may lead to fatty acids reaching the colon, thus free fatty acids in the colon may compete with oxalate to bind calcium. As a result, an increased levels of free oxalate might be reabsorbed via colonocytes and excreted in the urine, leading to oxalate urolithiasis [31]. In general, IBD represents a newly appreciated cause for the development of urolithiasis which may relate to gut-kidney axis disruption.uACR and eGFRcrea are the two key markers for CKD, and urolithiasis is a risk factor for CKD. However, our MR study has demonstrated that IBD is not the cause for increased levels of eGFRcrea and risk for CKD, despite the relations that UC and CD affect the levels of uACR and the risk of urolithiasis, separately. A single-cohort study showed that renal dysfunction is a rare complication (2%) in CD, and recurrent urolithiasis appears to be the primary causative factor [32]. A case–control study demonstrated that the frequency of renal dysfunction in IBD inpatients was 15.9% [33], this high prevalence of renal dysfunction may be associated with severity of IBD inpatients. Moreover, a recent study of over 80,000 individuals showed that IBD is correlated with an increased risk of CKD, and exposure to 5-aminosalicylates or methotrexate was not associated with the change in eGFRcrea [5]. A population-based study conducted across Korea showed that patients with CD had a substantially higher risk of ESRD in comparison with matched controls (HR = 6.33). Conversely, there was no significant difference in the risk of ESRD observed between UC and control groups [34]. GWAS data typically involves exposures and outcomes that are measured at a single timepoint. The interpretation of the causal estimates assumes that the effect of the genetic instruments on IBD remains stable over time. Nonetheless, the etiology of IBD involves a complex interaction between the genetic, the immune responses and environmental factors, thus environmental or epigenetic influences may alter the correlation between a genetic instrument and the exposure throughout the lifespan. In addition, our MR study cannot entirely exclude the hypothesis that IBD is the cause of the elevated levels of eGFRcrea and risk of CKD, since the disease activity and disease duration of IBD have not been extensively studied.

To the best of our knowledge, this is the first study to perform a MR analysis to address causality between IBD (including UC and CD) and kidney function/disease (including eGFRcrea, uACR, CKD, urolithiasis, and IgA nephropathy) using large-scale GWAS data. Importantly, our results show that UC increases the levels of uACR, and CD increases the risk of urolithiasis. The current study, however, has several limitations. First, the enrolled individuals were almost European, thus the causal effect of IBD on the levels of uACR and the risk of urolithiasis in other ethnic populations remains unknown. Second, the GWAS of IgA nephropathy were from a trans-ethnic population (73% European and 27% East Asian). Hence, we need to cautiously interpret the conclusion that there is no causal relationship between IBD and IgA nephropathy. Third, our findings only report alterations in kidney function and increased risk of urolithiasis in patients with IBD, thus further investigations are required to address underlying mechanisms. Notably, microphysiological systems could be an ideal approach to investigating the role of the gut-kidney axis in IBD [35]. Besides, IBD drug-induced nephrotoxicity should be taken into consideration in clinical practice of IBD as described in previous studies.

In conclusion, UC and CD affect the levels of uACR and increase the risk of urolithiasis, separately. The renal involvement in patients with IBD can be categorized into secondary diseases caused by IBD, co-occurring disease with IBD, and IBD drug-induced nephrotoxicity. As the rate of renal involvement in patients with IBD may be currently underestimated or even overlooked in clinics, monitoring of kidney function should be considered in clinical practice in patients with IBD.

## Supplementary Information

**Additional file 1: Table S1.** MR estimates from different methods of assessing the causal effect of IBD on kidney function. **Figure S1**. Non-significant Mendelian randomization association between UC and kidney function. (A) Scatter plot for genetically predicted UC on eGFRcrea; (B) Scatter plot for genetically predicted UC on CKD; (C) Scatter plot for genetically predicted UC on IgA nephropathy; (D) Scatter plot for genetically predicted UC on urolithiasis; UC, ulcerative colitis; eGFRcrea, estimated glomerular filtration rate from serum creatinine; CKD, chronic kidney disease; SNP, single nucleotide polymorphism. **Figure S2**. Non-significant Mendelian randomization association between CD and kidney function. (A) Scatter plot for genetically predicted CD on eGFRcrea; (B) Scatter plot for genetically predicted CD on uACR; (C) Scatter plot for genetically predicted CD on IgA nephropathy; (D) Scatter plot for genetically predicted CD on CKD; CD, Crohn’s disease; eGFRcrea, estimated glomerular filtration rate from serum creatinine; uACR, urine albumin to creatinine ratio; CKD, chronic kidney disease; SNP, single nucleotide polymorphism. **Figure S3**. Leave-one-out sensitivity analysis for UC on kidney function. (A) UC on eGFRcrea; (B) UC on CKD; (C) UC on IgA nephropathy; (D) UC on urolithiasis; UC, ulcerative colitis; eGFRcrea, estimated glomerular filtration rate from serum creatinine; CKD, chronic kidney disease. **Figure S4**. Leave-one-out sensitivity analysis for CD on kidney function. (A) CD on eGFRcrea; (B) CD on CKD; (C) CD on uACR; (D) CD on IgA nephropathy. CD, Crohn’s disease; eGFRcrea, estimated glomerular filtration rate from serum creatinine; uACR, urine albumin to creatinine ratio; CKD, chronic kidney disease.

## Data Availability

The summary-level GWAS data correlated with IBD were obtained from European-ancestry participants from the International Inflammatory Bowel Disease Genetics Consortium (https://www.ibdgenetics.org/). The summary-level GWAS data for kidney function were obtained from CKDGen Consortium (https://ckdgen.imbi.uni-freiburg.de/). The summary-level GWAS data for urolithiasis were obtained from the FinnGen consortium, Freeze 7 (https://r7.finngen.fi/pheno/N14_UROLITHIASIS). The summary-level GWAS data for IgA nephropathy were obtained from the meta-analysis of UK-biobank, FinnGen, and Biobank Japan (https://www.ebi.ac.uk/gwas/studies/GCST90018866).
