# Supplementary material for: Inflammatory bowel disease increases the levels of albuminuria and the risk of urolithiasis: a two-sample Mendelian randomization study
Source: Eur J Med Res. 2023 May 12;28:167. doi: 10.1186/s40001-023-01128-0 (PMC10176914; doi:10.1186/s40001-023-01128-0)
Supplement: Supplementary file 1 — Additional file 1: Table S1. MR estimates from different methods of assessing the causal effect of IBD on kidney function. Figure S1. Non-significant Mendelian randomization association between UC and kidney function. (A) Scatter plot for genetically predicted UC on eGFRcrea; (B) Scatter plot for genetically predicted UC on CKD; (C) Scatter plot for genetically predicted UC on IgA nephropathy; (D) Scatter plot for genetically predicted UC on urolithiasis; UC, ulcerative colitis; eGFRcrea, estimated glomerular filtration rate from serum creatinine; CKD, chronic kidney disease; SNP, single nucleotide polymorphism. Figure S2. Non-significant Mendelian randomization association between CD and kidney function. (A) Scatter plot for genetically predicted CD on eGFRcrea; (B) Scatter plot for genetically predicted CD on uACR; (C) Scatter plot for genetically predicted CD on IgA nephropathy; (D) Scatter plot for genetically predicted CD on CKD; CD, Crohn’s disease; eGFRcrea, estimated glomerular filtration rate from serum creatinine; uACR, urine albumin to creatinine ratio; CKD, chronic kidney disease; SNP, single nucleotide polymorphism. Figure S3. Leave-one-out sensitivity analysis for UC on kidney function. (A) UC on eGFRcrea; (B) UC on CKD; (C) UC on IgA nephropathy; (D) UC on urolithiasis; UC, ulcerative colitis; eGFRcrea, estimated glomerular filtration rate from serum creatinine; CKD, chronic kidney disease. Figure S4. Leave-one-out sensitivity analysis for CD on kidney function. (A) CD on eGFRcrea; (B) CD on CKD; (C) CD on uACR; (D) CD on IgA nephropathy. CD, Crohn’s disease; eGFRcrea, estimated glomerular filtration rate from serum creatinine; uACR, urine albumin to creatinine ratio; CKD, chronic kidney disease. [file 40001_2023_1128_MOESM1_ESM.docx]

**Table S1.** MR estimates from different methods of assessing the causal effect of IBD on kidney function.

|  | **No. of SNP^*^** | **IVW**  ***β* or OR (95%CI)** | ***P*** | **WM**  ***β* or OR (95%CI)** |  | ***P*** | **MR-Egger**  ***β* or OR (95%CI)** | ***P*** |
| --- | --- | --- | --- | --- | --- | --- | --- | --- |
| UC and eGFRcrea | 73 | –0.000495  (–0.001353, 0.000363) | 0.26 | –0.000701  (–0.001912, 0.00051) |  | 0.26 | 0.0009  (–0.001039, 0.003003) | 0.3 |
|  |  |  |  |  |  |  |  |  |
| UC and uACR | 75 | 0.007924  (0.002993, 0.012856) | **0.002** | 0.00683  (–0.000073, 0.013733) |  | 0.052 | 0.005283  (–0.006313, 0.016878) | 0.40 |
|  |  |  |  |  |  |  |  |  |
| UC and urolithiasis | 76 | 1.005518  (0.966641, 1.045957) | 0.78 | 1.01952  (0.969545, 1.07207) |  | 0.45 | 1.032088  (0.940552, 1.132534) | 0.51 |
|  |  |  |  |  |  |  |  |  |
| UC and IgA nephropathy | 78 | 1.001003  (0.985439, 1.035242) | 0.43 | 1.019068  (0.984109, 1.055268) |  | 0.29 | 1.010066  (0.952152, 1.071503) | 0.74 |
|  |  |  |  |  |  |  |  |  |
| UC and CKD | 80 | 1.005527  (0.983058, 1.028306) | 0.64 | 0.975207  (0.944667, 1.006735) |  | 0.12 | 1.002915  (0.95027, 1.058476) | 0.92 |
|  |  |  |  |  |  |  |  |  |
| CD and eGFRcrea | 93 | 0.000184  (–0.000739, 0.001108) | 0.70 | –0.00068  (–0.001778, 0.000418) |  | 0.22 | 0.000459  (–0.001978, 0.002895) | 0.71 |
|  |  |  |  |  |  |  |  |  |
| CD and uACR | 99 | 0.003361  (–0.001622, 0.008344) | 0.19 | 0.003306  (–0.002921, 0.009534) |  | 0.30 | 0.003021  (–0.010882, 0.016924) | 0.67 |
|  |  |  |  |  |  |  |  |  |
| CD and urolithiasis | 102 | 1.045481  (1.007904, 1.084459) | **0.017** | 1.012803  (0.964924, 1.063057) |  | 0.61 | 1.010317  (0.913279, 1.117665) | 0.84 |
|  |  |  |  |  |  |  |  |  |
| CD and IgA nephropathy | 104 | 1.002251  (0.981507, 1.023434) | 0.83 | 0.992097  (0.961875, 1.023269) |  | 0.62 | 0.986685  (0.931273, 1.045394) | 0.65 |
|  |  |  |  |  |  |  |  |  |
| CD and CKD | 106 | 1.017845  (0.999253, 1.036784) | 0.06 | 1.017273  (0.992478, 1.042686) |  | 0.17 | 1.005454  (0.956568, 1.056839) | 0.83 |

SNP^*^, MR analysis after removing all the outliers by MR-PRESSO; IBD, inflammatory bowel disease; UC, ulcerative colitis; CD, Crohn’s disease; eGFRcrea, estimated glomerular filtration rate from serum creatinine; uACR, urine albumin to creatinine ratio; CKD, chronic kidney disease; IVW, inverse variance weighted; WM, weighted median; OR, odds ratio.


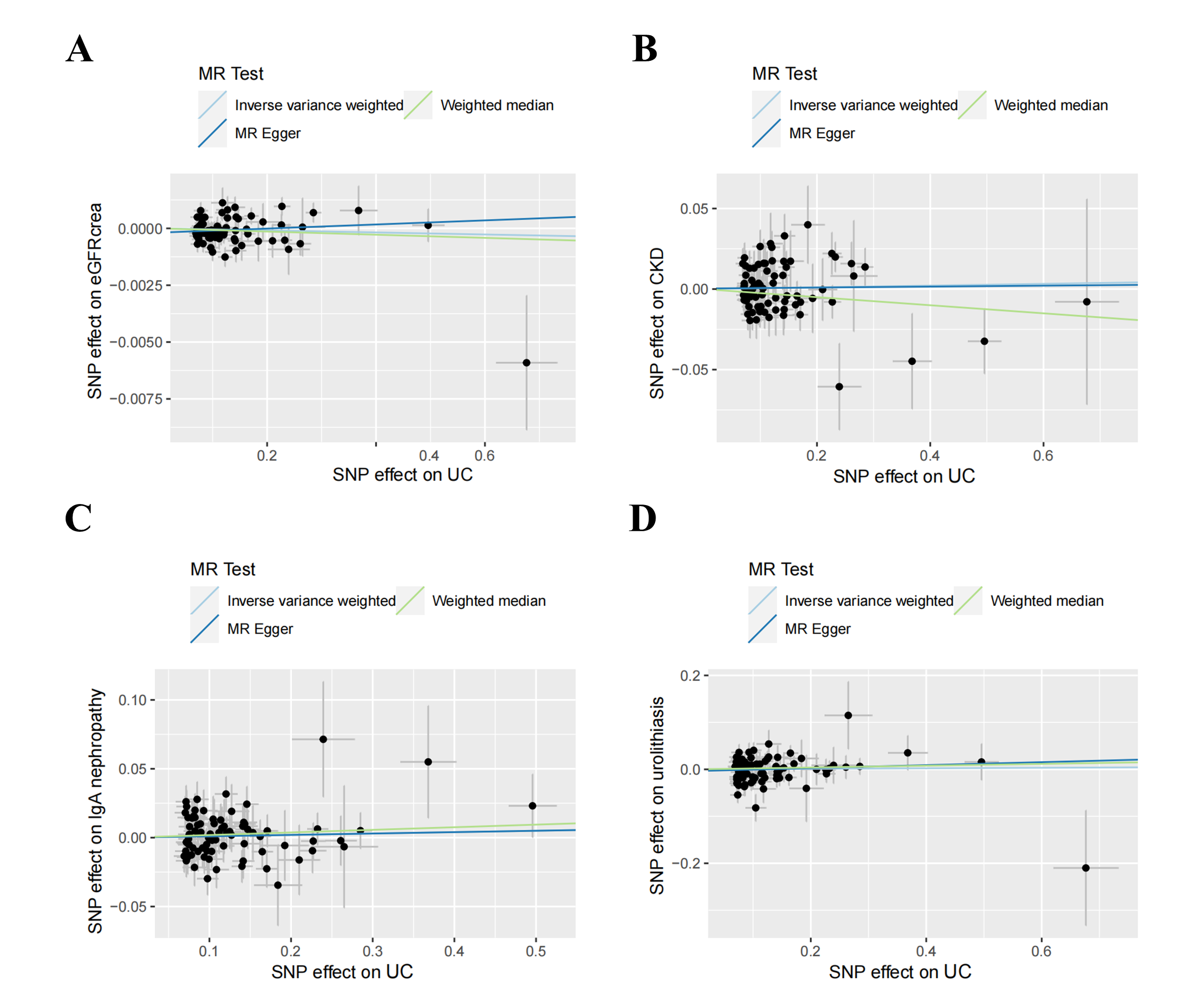


**Figure S1**. Non-significant Mendelian randomization association between UC and kidney function. (A) Scatter plot for genetically predicted UC on eGFRcrea; (B) Scatter plot for genetically predicted UC on CKD; (C) Scatter plot for genetically predicted UC on IgA nephropathy; (D) Scatter plot for genetically predicted UC on urolithiasis; UC, ulcerative colitis; eGFRcrea, estimated glomerular filtration rate from serum creatinine; CKD, chronic kidney disease; SNP, single nucleotide polymorphism.


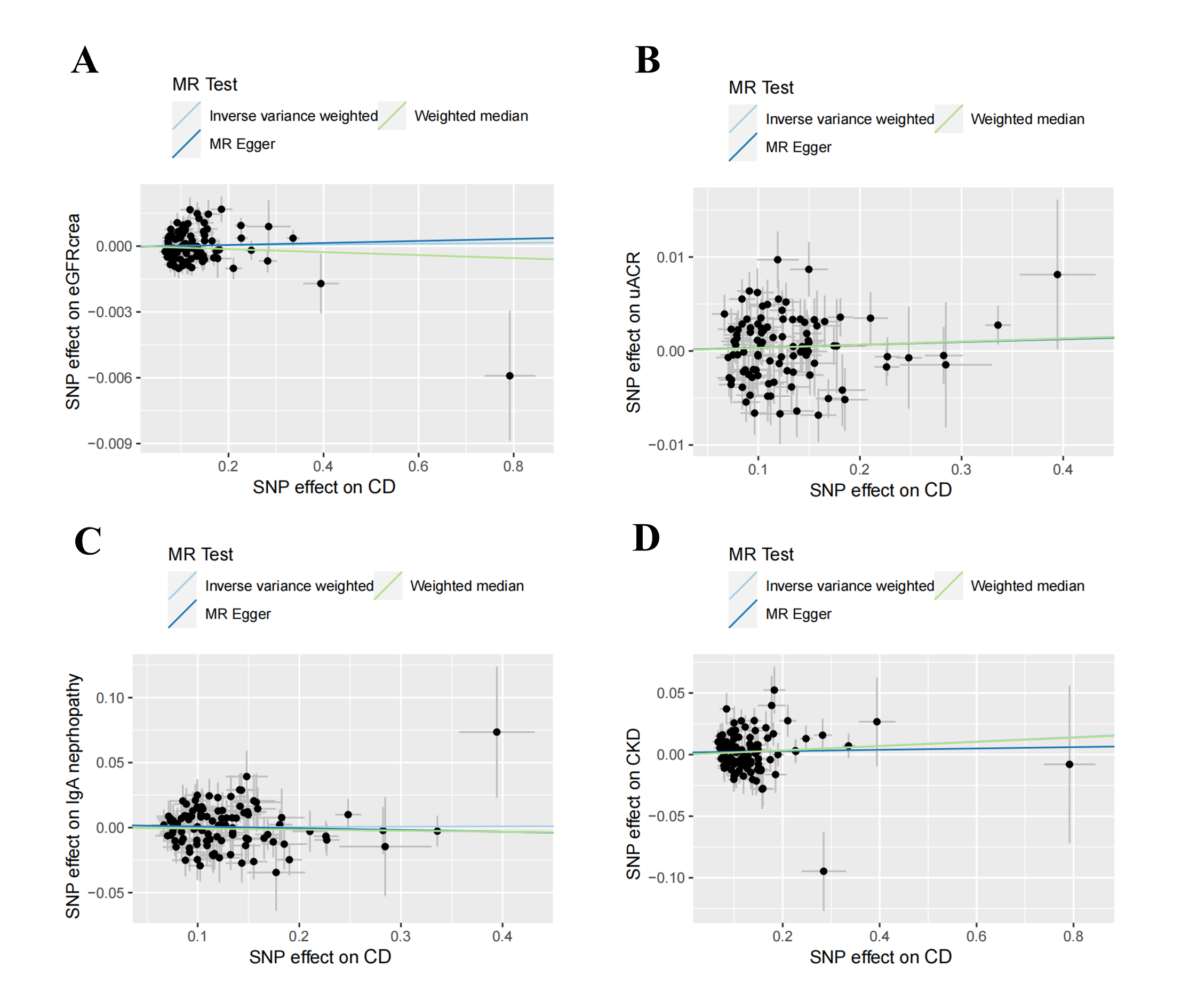


**Figure S2**. Non-significant Mendelian randomization association between CD and kidney function. (A) Scatter plot for genetically predicted CD on eGFRcrea; (B) Scatter plot for genetically predicted CD on uACR; (C) Scatter plot for genetically predicted CD on IgA nephropathy; (D) Scatter plot for genetically predicted CD on CKD; CD, Crohn’s disease; eGFRcrea, estimated glomerular filtration rate from serum creatinine; uACR, urine albumin to creatinine ratio; CKD, chronic kidney disease; SNP, single nucleotide polymorphism.


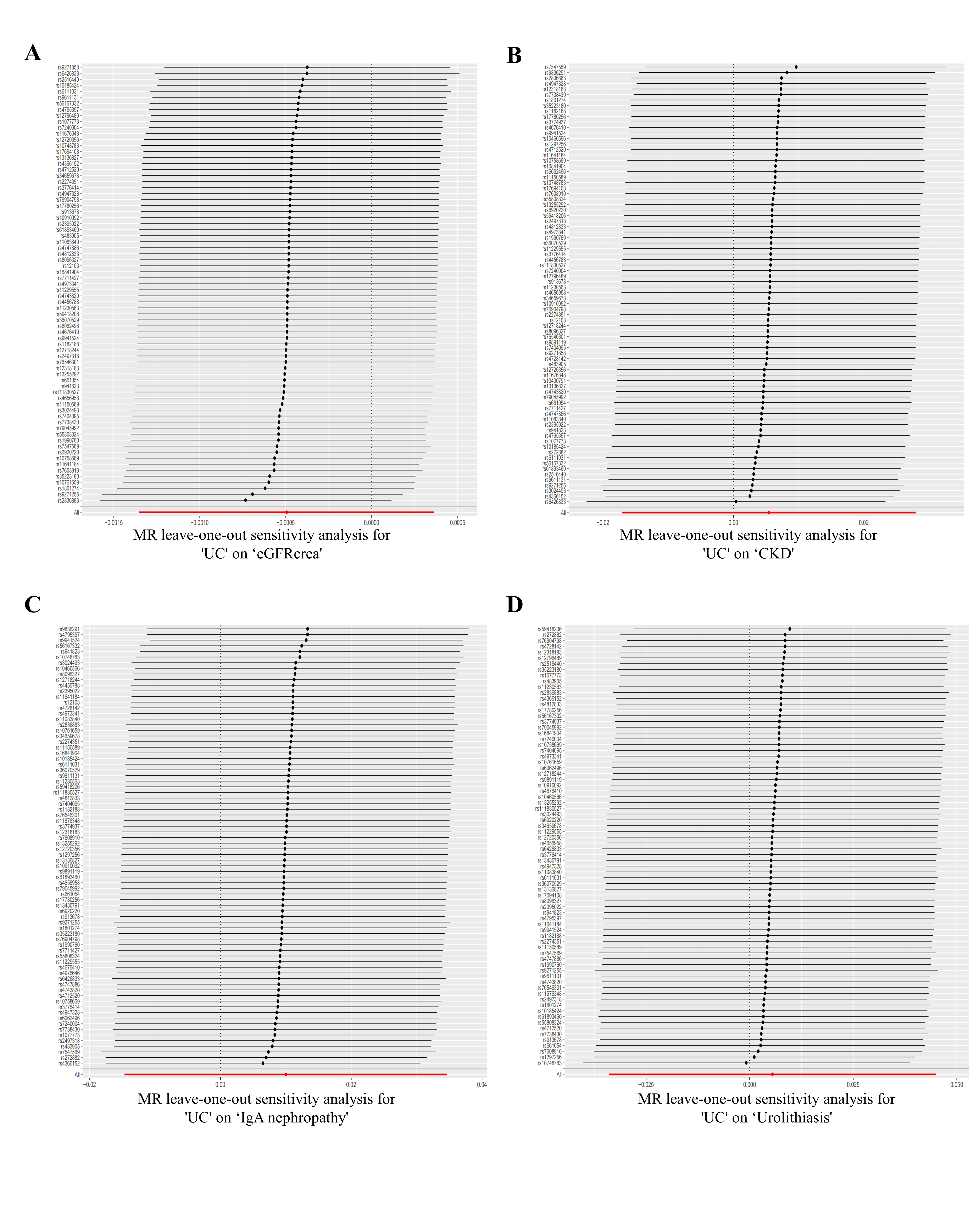


**Figure S3**. Leave-one-out sensitivity analysis for UC on kidney function. (A) UC on eGFRcrea; (B) UC on CKD; (C) UC on IgA nephropathy; (D) UC on urolithiasis; UC, ulcerative colitis; eGFRcrea, estimated glomerular filtration rate from serum creatinine; CKD, chronic kidney disease.


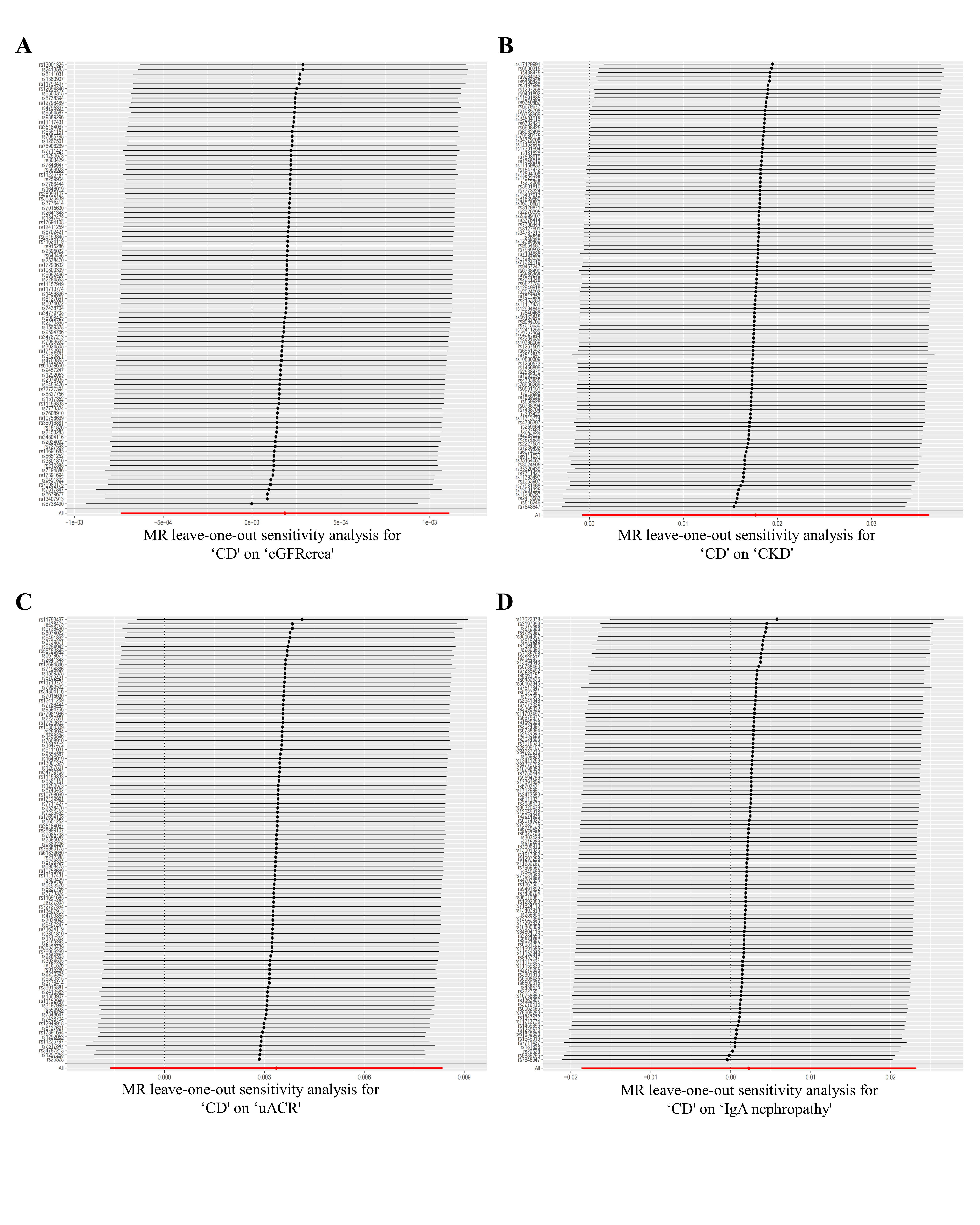


**Figure S4**. Leave-one-out sensitivity analysis for CD on kidney function. (A) CD on eGFRcrea; (B) CD on CKD; (C) CD on uACR; (D) CD on IgA nephropathy. CD, Crohn’s disease; eGFRcrea, estimated glomerular filtration rate from serum creatinine; uACR, urine albumin to creatinine ratio; CKD, chronic kidney disease.
